# Incidence, time to recovery and predictors among neonates admitted with respiratory distress to the neonatal intensive care unit at the University of Gondar Comprehensive Specialized Hospital, Northwest Ethiopia, 2021

**DOI:** 10.1371/journal.pone.0278887

**Published:** 2022-12-15

**Authors:** Engidaw Fentahun Enyew, Desalegn Anmut Bitew, Abebaw Addis Gelagay

**Affiliations:** 1 Department of Anatomy, School of Medicine, College of Medicine and Health Science, University of Gondar, Gondar, Ethiopia; 2 Department of Reproductive Health, Institute of Public Health, College of Medicine and Health Science, University of Gondar, Gondar, Ethiopia; Kaohsuing Medical University Hospital, TAIWAN

## Abstract

**Background:**

One of the major factors contributing to newborn morbidity and mortality across the globe is respiratory distress. In resource-constrained developing nations like Ethiopia, it is a significant issue. Depending on the quality of the care provided, the incidence and time to recovery may differ amongst medical facilities. However, Ethiopia still lacks appropriate data on the incidence and time to recovery from respiratory distress.

**Objective:**

The aim of the study was to assess the incidence, time to recovery, and predictors among neonates admitted with respiratory distress in the neonatal intensive care unit at the University of Gondar Comprehensive Specialized Hospital.

**Methods:**

An institution-based retrospective follow-up study design was conducted among 452 neonates with respiratory distress. Data were collected using a data extraction checklist from the medical registry. The extracted data were entered into EPI INFO version 7.2.1.0 and then exported to STATA version 14 for analysis. The median time to recovery, the Kaplan Meier curve, and the log-rank test was computed. Both bi-variable and multivariable Cox regression models were applied to analyze the data. p-value ≤ 0.05 was considered statistically significant.

**Results:**

Of all respiratory distressed neonate,311 were recovered. The overall incidence rate of neonates admitted with from respiratory distress was 11.5 per 100-neonate day (95% CI: 10.30–12. 87) with 2,703-person day observation and the median time to recovery from respiratory distress was 7 days with (IQR = 3–13 days). Predictors of time to recovery from respiratory distress were very low birth weight (AHR = 0.17, 95% CI: 0.08–0.41), low birth weight (AHR = 0.50, 95% CI: 0.31–0.81), very preterm (AHR = 0.42,95% CI:0.20–0.89), sepsis (AHR = 0.50 95% CI: 0.38–0.65), hypothermia (AHR = 0.61, 95% CI: 0.39–0.81), and Apgar scores less than seven at first (AHR = 0.35, 95% CI: 0.15–0.79) and fifth minute (AHR = 0.45, 95% CI: 0.20–0.97).

**Conclusion:**

The incidence and time to recovery in this study were discreetly acceptable as compared to previous study. The aforementioned predictors could be used to identify neonates with respiratory distress who are at risk of developing a long-term illness and guide prompt referral to hospitals. This will also provide clinicians with prognostic information, as longer recovery times have economic and social implications in resource limited countries like Ethiopia.

## Introduction

Respiratory distress (RD) in neonates is a significant cause of morbidity and mortality and a common condition requiring admission to the neonatal intensive care unit (NICU) [1, 2].

After birth or throughout the change from foetal to neonatal life, RD is a significant issue for neonates [3, 4]. For a healthy transition from foetal to newborn life, rapid physiologic changes in the cardiorespiratory systems are required. Because of these changes, gas exchange is redirected from the placenta to the lungs, and normal breathing requires the replacement of alveolar fluid with air [4]. Despite worsening in developing countries, it is a significant global source of neonates morbidity and mortality [5, 6].

Around 10% of newborns require some assistance to start breathing after they are born, and up to 1% may need significant resuscitation, according to the American Academy of Pediatrics [7]. Neonates with respiratory distress are 2–4 times more likely to die than neonates who do not have respiratory distress [8].

Respiratory distress affects 2.2% to 7.6% of all term deliveries worldwide, and 50%, 75%, and 90% of newborns born at 30 weeks, 28 weeks, and 26 weeks, respectively [9]. Significant respiratory morbidity affects 29% of late preterm newborns and 15% of term infants admitted to the NICU [3]. This is much more common in newborns born before 34 weeks of gestation [10]. Furthermore, it appears that RD morbidity is higher in low-resource settings than in high-income countries [2].

There are recognized causes of RD in both developed and developing nations, such as preterm, low first and fifth-minute Apgar scores, meconium aspiration syndrome, caesarean birth, gestational diabetes, maternal chorioamnionitis, sepsis and premature rupture of membranes (PROM) [11–15].

RD, regardless of the cause, can lead to respiratory failure, cardiopulmonary arrest, and even death if not recognized and treated promptly. As a result, any health care worker caring for newborn neonates must be able to recognize the signs and symptoms of RD, differentiate the various causes, and initiate management strategies to avoid significant complications or death. As a result, neonates who require critical medical attention are typically admitted to the NICU. These infants are typically preterm, have a low birth weight, or have serious medical conditions such as RD [16].

The Sustainable Development Goals, as well as other policies, strategies, and programs, work to prevent and care for preterm neonates and their birth outcomes, including RD [17, 18]. Despite these efforts, RD remains one of the main causes of neonatal morbidity and mortality in Ethiopia [18, 19], resulting in rising neonatal care costs. However, few studies on RD in these areas have been conducted in developing countries, including Ethiopia. As a result, the aim of this study was to determine the incidence, time to recovery, and predictors of respiratory distress in neonates admitted to the NICU in the University of Gondar Comprehensive Specialized Hospital (UOGCSH).

## Methods

### Study area and period

The study was conducted in UOGCSH Northern Ethiopia, which is located approximately 728 km away from the capital city, Addis Ababa, 175 km from the Regional Capital, Bahir Dar. The hospital serves a population of over 7 million people in northwest Ethiopia. The NICU is a unit within the department of pediatrics and child health that provides an intensive care unit for neonates and has a capacity of approximately 40 beds at any given time. The space also accommodates invasive and non-invasive ventilations. On average, 845 births occur in the hospital each month from gestational ages of twenty-eight weeks and above, which are considered viable. The study was conducted from 01/01/2021 to 30/06/2021.

### Study design

An institution-based retrospective study was conducted among neonates admitted with respiratory distress in the NICU.

### The source and study population

All neonates admitted with respiratory distress to the NICU at UOGCSH were the source populations, and all neonates admitted with respiratory distress during the study period were the study populations.

### Eligibility criteria

All neonates admitted with the diagnosis of RD in UOGCSH during the study period were included in the study. Neonates who recorded the date of admission/date of discharge and had missing charts were excluded from the study

### Variables of the study

#### Dependent variable

Time to recovery from RD in (days).

#### Independent variables

**Sociodemographic factors**; residence, sex, birth weight of the neonate and gestational age.

**Obstetric and related factors:** parity, mode of delivery, place of delivery, multiple pregnancies, PROM, preeclampsia, and placental abruption.

**Medical disorders in mother**: gestational hypertension, maternal diabetes mellitus and human immunodeficiency virus/acquired immunodeficiency syndrome (HIV/AIDS).

**Neonatal outcome condition**: birth asphyxia, Apgar score, sepsis, jaundice, hypothermia, hypoglycemia, meconium aspiration and congenital anomalies.

### Operational definitions

#### Recovery

If a neonate was recovered from RD after completing treatment based on physician diagnosis.

#### Time to recovery

A period of time between the neonate’s admission by RD and his or her discharge while the neonate is recovered. It was calculated by subtracting the date of admission from the date of discharge (time in days until recovery/discharge).

#### Censored

It refers to a neonates referred, died, transferred or defaulted from treatment.

#### Respiratory distress

The presence of two or more of the following signs: an abnormal respiratory rate (tachypnea *>*60 breaths/min, bradypnea *<*30 breaths/minute, respiratory pauses, or apnea) or signs of labored breathing (expiratory grunting, nasal flaring, intercostal recessions, xyphoid recessions), with or without cyanosis [12].

### Sample size determination and sampling technique

The sample size was determined by a power approach using the sample size determination formula for survival analysis [20]:

$$A={\left(Z_{\alpha /2}+Z_{\beta }\right)}^{2}\;B={\left(log\;(RH)\right)}^{2}p_{1}p_{2}$$

Total sample size needed (n) = (A/B)/e where n is the required sample size, *Z*_*α*/2_ is the critical value of the standard normally distributed variable at the 5% significance level (1.96), *Z_β_* is the critical value of the standard normally distributed variable at the 20% significance level or type two error (0.8416), RH is the log (hazard ratio), *p*_1_ is the proportion of patients in the first category, and *p*_2_ is the proportion of patients in the second category [20] The proportion of recovered RD patients in this case was e = 0.429, based on a previous study conducted in a black lion specialized hospital [21]. Using the above formula, the sample size was calculated taking into account the following factors: PROM, sex, sepsis, maternal diabetes. Finally, the maximum appropriate sample size is obtained for PROM, which is 452.After determining the sampling fraction (k = 3), the neonates’ cards were accessed using the systematic random sampling technique, and the first card was drawn using the lottery method.

### Data collection tool and procedure

The information was gathered using a checklist adapted from RD neonatal charts and similar studies (22, 23). The data collection checklist was written in English. The checklist includes information on sociodemographic characteristics of neonates with RD, as well as the mother’s maternal medical condition, neonatal medical condition, and obstetric- and gynecological-related predictors. Before collecting data, the records were reviewed, and RD neonatal cards were identified by their medical registration number. After one day of training, two BSc nurse data collectors were supervised by one BSc nurse supervisor. The data was then extracted using a structured and pretested data extraction checklist.

### Data quality control

Designing appropriate data abstraction tools ensured data quality. One day of training was also provided for both data collectors and supervisors on the data abstraction tool and data collection process. The supervisors and principal investigator closely monitored the day-to-day data collection process to ensure the checklist was complete and consistent. The data was evaluated daily for completeness, and any difficulties encountered during data collection were addressed accordingly. Finally, the supervisor and investigator double-checked all collected data for completeness and consistency during data management, storage, and analysis.

### Data processing and analysis

Data were collected using a semi-structured checklist, and each questionnaire was checked for completeness of data, assigned a code, entered into EPI INFO 7.2, and exported to Stata14 statistical software for analysis. Prior to analysis, the data were cleaned, and missing values were handled by revising the original coded questionnaire. The median time to recovery, Kaplan Meier curve, and log-rank test were computed. The proportional hazard assumption was validated graphically as well as through Schoenfeld residual global tests. The bivariable and multivariable Cox regression models were used to describe the association between the dependent and independent variables, as well as independent predictors of time to recovery. To control for potential confounding covariates at the same time, covariates with a P-value of 0.05 in bivariate analysis were entered into a multivariable regression analysis. The Cox Snell residual test was used to evaluate the model’s goodness of fit. The Crude Hazard Ratio (CHR) and Adjusted Hazard Ratio (AHR) were used to assess the strength of association between the independent and dependent variables. Overall, a P-value ≤ 0.05 was considered statistically significant, with their respective 95% confidence intervals.

### Ethical considerations

Ethical clearance was obtained from the University of Gondar, Institute of Public Health Ethical Review Committee (Ref No/IPH/1504/2013 E.C.). The members of the ethical review Committee wrote letters of cooperation to UOGCSH, and permission was later obtained from the clinical director, department head and card room head at the hospital. The patients will not be harmed because the study was carried out using appropriate information from their medical charts. The name or any other identifying information was not recorded on the checklist, and all information obtained from the chart was strictly confidential and kept in a secure location. Following these approvals, access to the medical charts was granted, and we did our best to maintain participant confidentiality by storing them in a file cabinet and keeping them in a keyed and locked.

## Results

### Sociodemographic and clinical characteristics of neonates

A total of 452 neonatal charts were reviewed and included in the final analysis. More than 90% of the neonates were admitted within the first few weeks of life, and 251 (55.5%) of the study participants were males. In terms of gestational age, 90 (19.91%) were very preterm (28–31 weeks), 107 (23.67%) were late preterm (32–36 weeks), 229 (50.67%) were term (37–41 weeks), and 26 (5.75%) were post term (> = 42 weeks). Similarly, neonates with normal birth weight made up half (50.67%) of the study participants. Neonatal sepsis (299, 66.15%) was diagnosed in nearly two-thirds of neonates admitted to the NICU, followed by hypoglycemia (138, 30.53%), meconium aspiration syndrome (109, 24.12%), perinatal asphyxia (87,19.25%), hypothermia (64, 14.16%), and jaundice (40, 8.85%). Furthermore, in the first and fifth minutes, 114 (25.22%) and 104 (23.01%) neonates had Apgar scores less than seven (**Table 1**).

**Table 1:** Sociodemographic and clinical characteristics of neonates admitted to the NICU in UOGCSH, 2021 (n = 452).

Variables and categoris	Frequency	Percent (%)
Ageof neonate atadmission in days		
0–6	415	91.8
7–28	37	8.2
Sex of neonate		
Female	201	44.47
Male	251	55.53
Birth weight ingrams		
Very low birth weight	50	11.06
Low birth weigh	155	34.29
Normal birth weight	229	50.67
Over birth weight	18	3.98
Gestational age in weeks		
Very preterm	90	19.91
prterm	107	23.67
Term	229	50.67
Post term	26	5.75
Perinatal asphyxia		
No	365	80.75
Yes	87	19.25
Sepsis		
No	153	33.85
Yes	299	66.15
Miconioum aspiration syndrome		
No	343	75.88
Yes	109	24.12
Jaundice		
No	412	91.15
Yes	40	8.85
Hypoglycemia		
No	314	69.47
Yes	138	30.53
Hypothermia		
No	388	85.84
Yes	64	14.16
APGAR score at first minute		
<7	114	25.22
≥7	338	74.78
APGAR score at fifth minute		
<7	104	23.01
≥7	348	76.99

### Sociodemographic and obstetric characteristics of mothers

The majority (81.2%) of all mothers of neonates enrolled in this study were between the ages of 20 and 34, with a median age of 27 years with IQR (23, 30). More than half of the mothers (60.84%) lived in cities. Concerning the mothers’ health, 42 (9.29%) had medical problems, 16 (3.54%) had HIV/AIDS, 14 (3.10%) had diabetes, 12 (2.65%) had hypertension, and 83 (18.36%) had pregnancy-related problems. PROM, preeclampsia, placental abruption, and others accounted for 30 (6.64%), 20 (4.42%), 17 (3.76%), and 16 (3.54%) of these cases, respectively. According to the findings of this study, nearly two-thirds of neonates (292, 64.6%) were born to mothers who had ANC follow-up. Almost three-fourths of mothers had spontaneous vaginal deliveries and gave birth in the same hospital. On the other hand, 43 (9.51%) of mothers had multiple pregnancies (**Table 2**).

**Table 2:** Sociodemographic, medical and obstetric characteristics of mothers of neonates admitted to the NICU in UOGCSH, 2021 (n = 452).

Variables and Categories	Frequency	Percent
Age of the mother		
<20	25	5.53
20–34	367	81.20
≥35	60	13.27
Residence		
Rural	177	39.16
Urban	275	60.84
Presence of medical problems		
No	410	90.71
Yes	42	9.29
Types of medical problem HIV/AIDS	16	3.54
Diabetes mellitus	14	3.10
Hypertension	12	12.65
Presence pregnancy related problems		
No	369	81.64
Yes	83	18.36
PROM	30	6.64
Preeclampsia	20	4.42
Abruption placenta	17	3.76
Others*	16	3.54
Parity		
1–4	403	89.16
>4	49	10.84
ANC follow up at least four times		
No	160	35.40
Yes	292	64.60
Mode of delivery		
Spontaneous vaginal delivery	322	71.24
Cesarean section	130	28.76
Place of delivery		
Referred from other health facilities	117	25.88
Delivered at the same Hospital	335	74.12
Multiple pregnancy		
No	409	90.49
Yes	43	9.51

(Others*) = placenta previa, antepartum hemorrhage

### The incidence rate and median time to recovery

**Fig 1:**
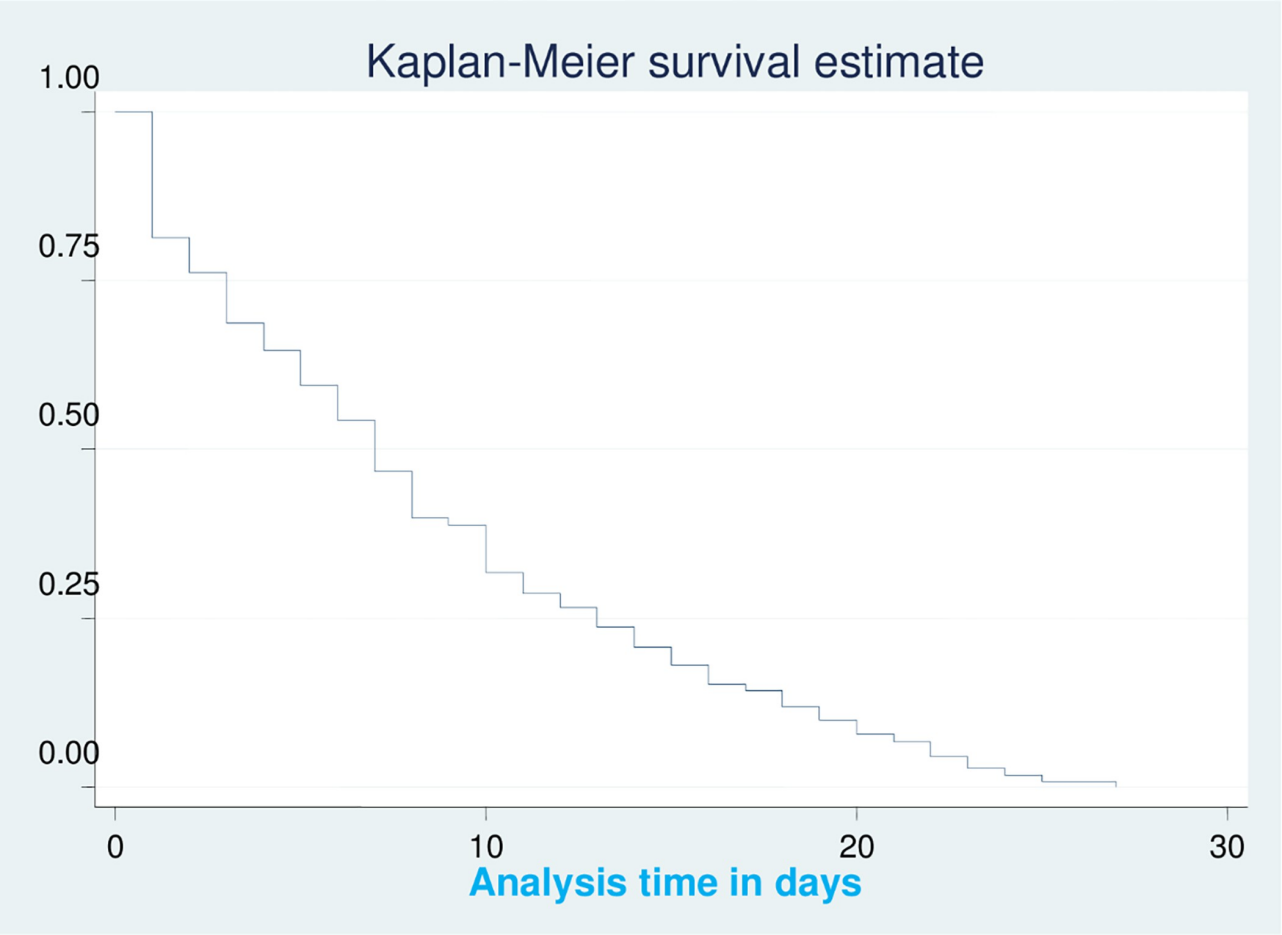
Kaplan–Meier survival estimates of the median time to recovery from RD of neonates admitted to the NICU in UOGCSH, 2021.

The neonates with RD were followed for a total of 2,703-neonate day observations. Approximately two-thirds of the neonates, 311 (68.6%), developed the event (recovered), while 141 (31.2%) neonates were censored and 74 (16.37%) died. The overall RD recovery rate was 11.5 per 100-person day (95% CI: (10.30, 12.87)). The overall median recovery time from RD (IQR) was 7 days (3, 13 days) (**Fig 1**).

### Comparison of survival status for different factors with the log rank test

**Fig 2:**
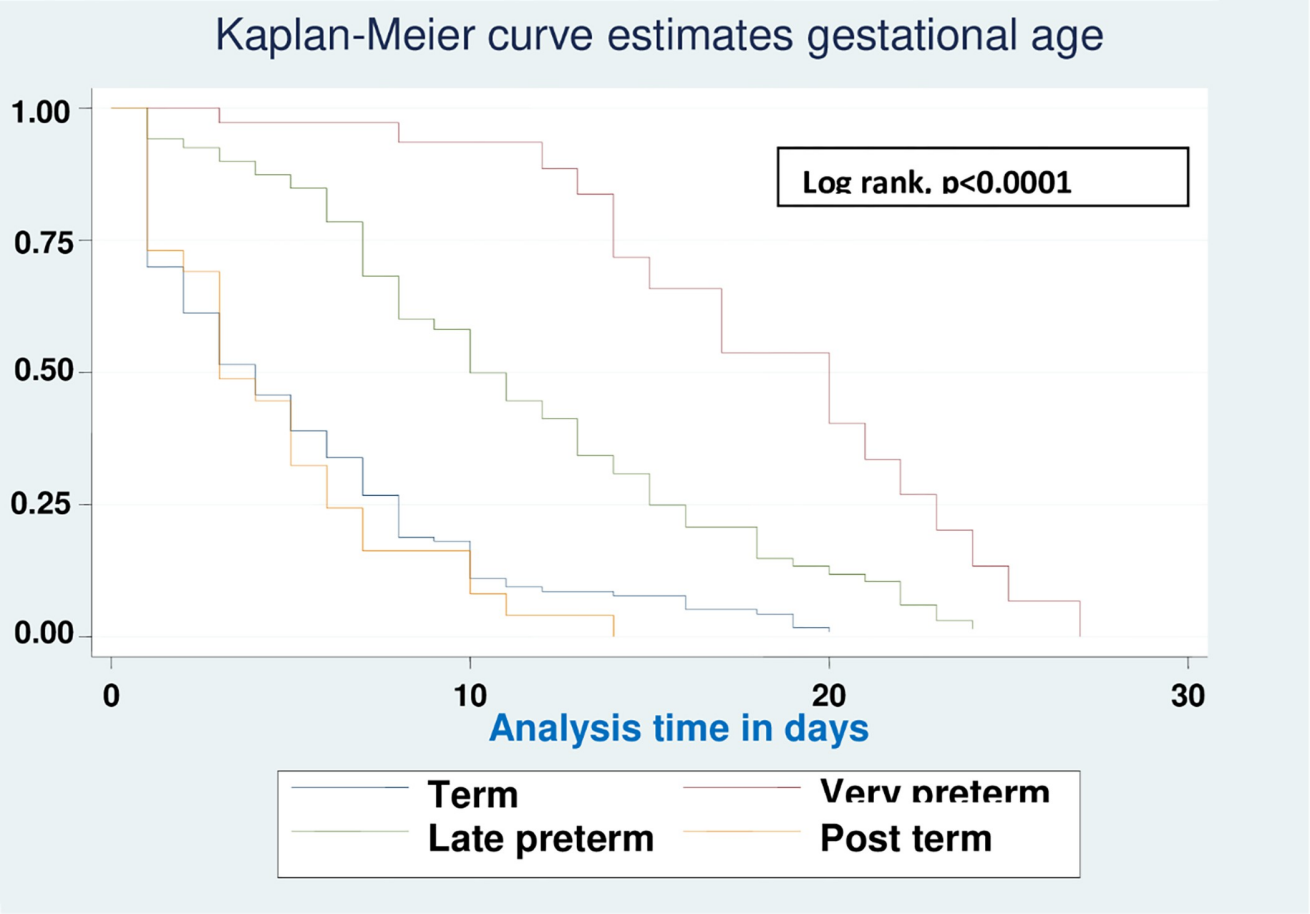
Kaplan-Meier curve of survival of neonates with RDS admitted in NICU by gestational age (A), birth weight (B), sepsis (C), hypothermia (D), Apgar scores for the first (E) and fifth minutes (F) at UOGCSH, 2021.

**Fig 3:**
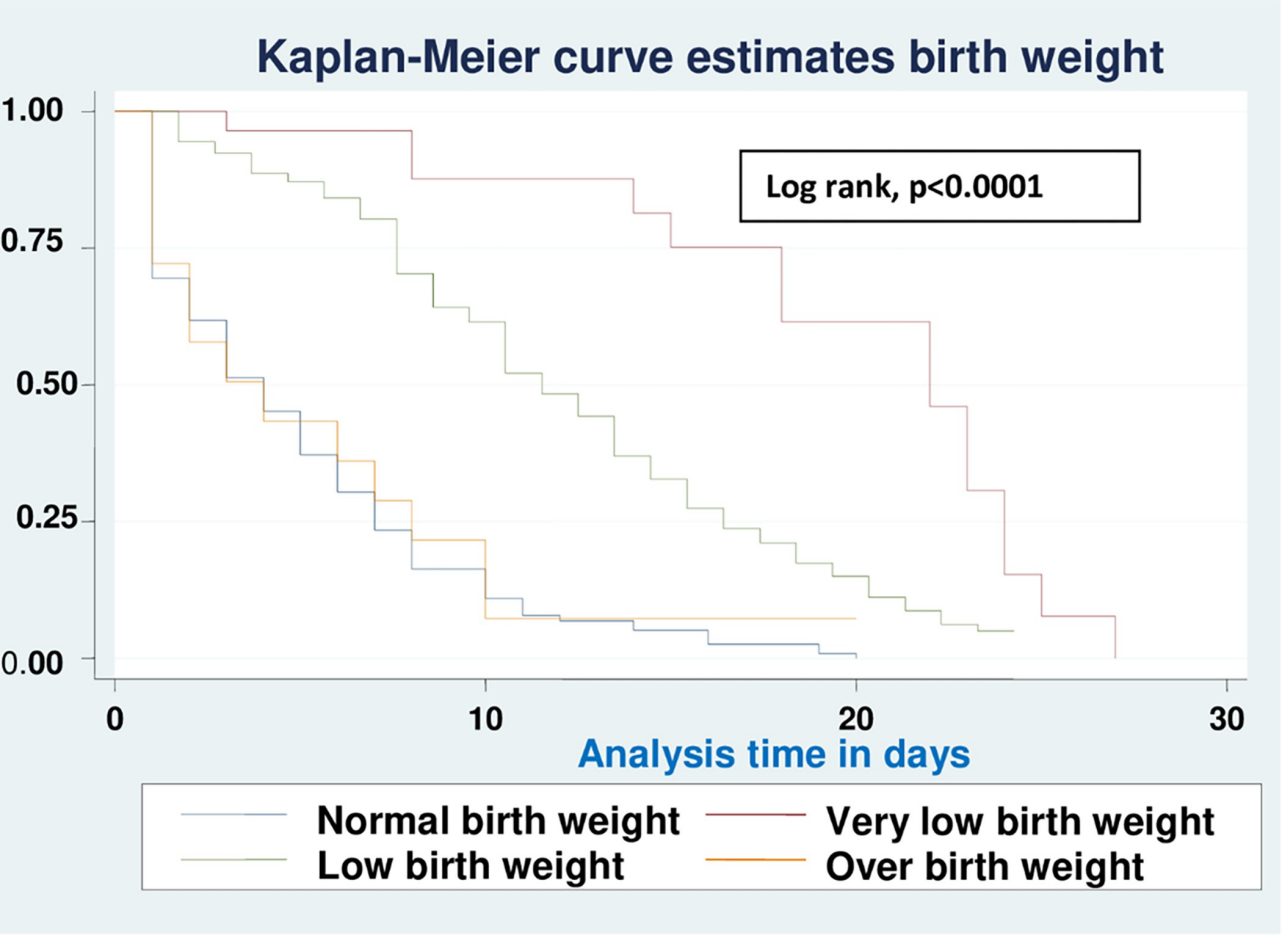
Cox-Snell Residual Graph for the goodness of model fitness that shows the hazard function follows the 45˚ closed to the baseline, in UOGCSH, 2021.

**Fig 4:**
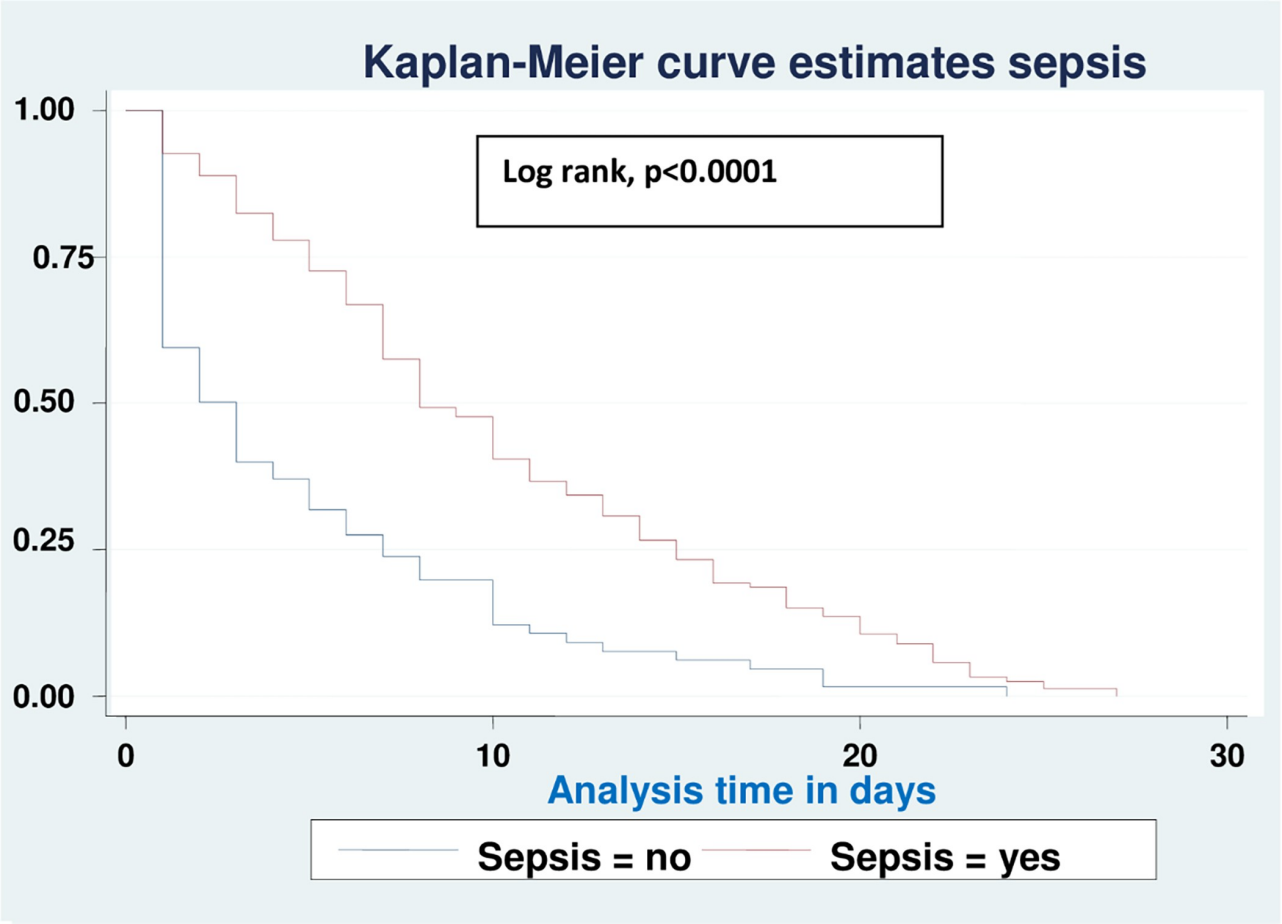
Comparison of Kaplan-Meier survival curves for sepsis with the log rank test of neonates with RD admitted to the NICU in UOGCSH, 2021.

**Fig 5:**
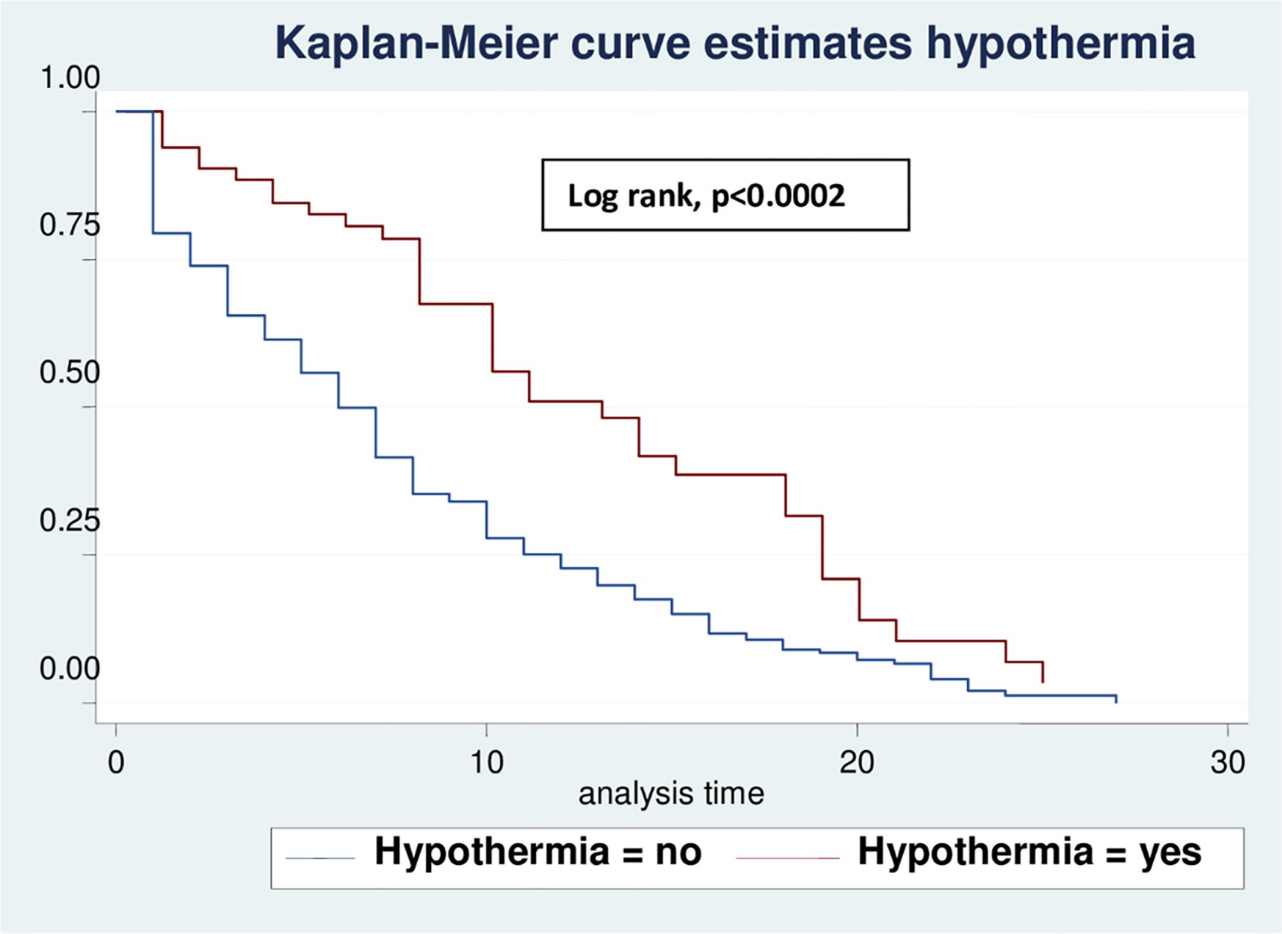
Comparison of Kaplan-Meier survival curves for hypothermia with the log rank test of neonates with RD admitted to the NICU in UOGCSH, 2021.

**Fig 6:**
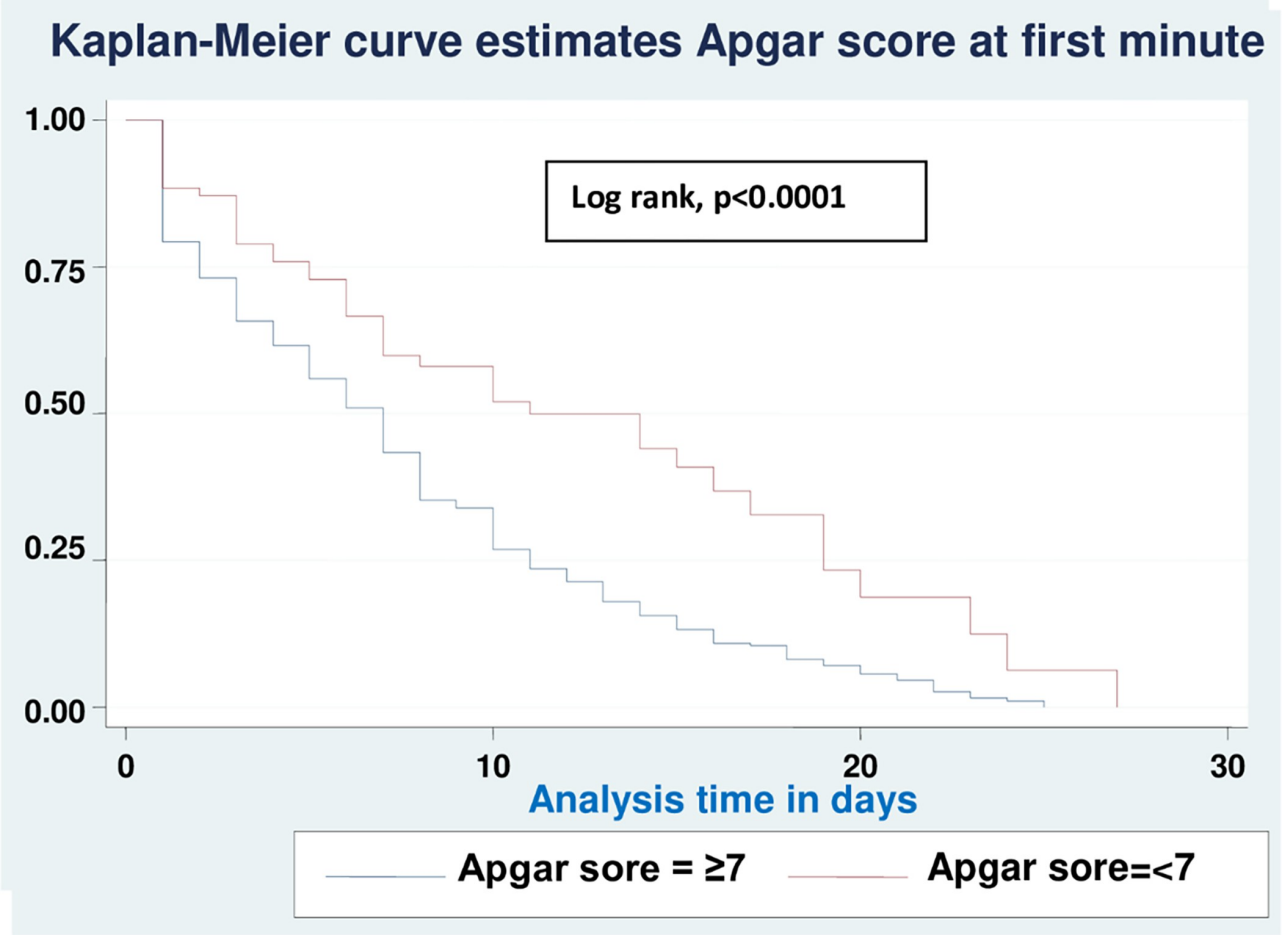
Comparison of Kaplan-Meier survival curves for Apgar score (1min) with the log rank test of neonates with RD admitted to the NICU in UOGCSH, 2021.

**Fig 7:**
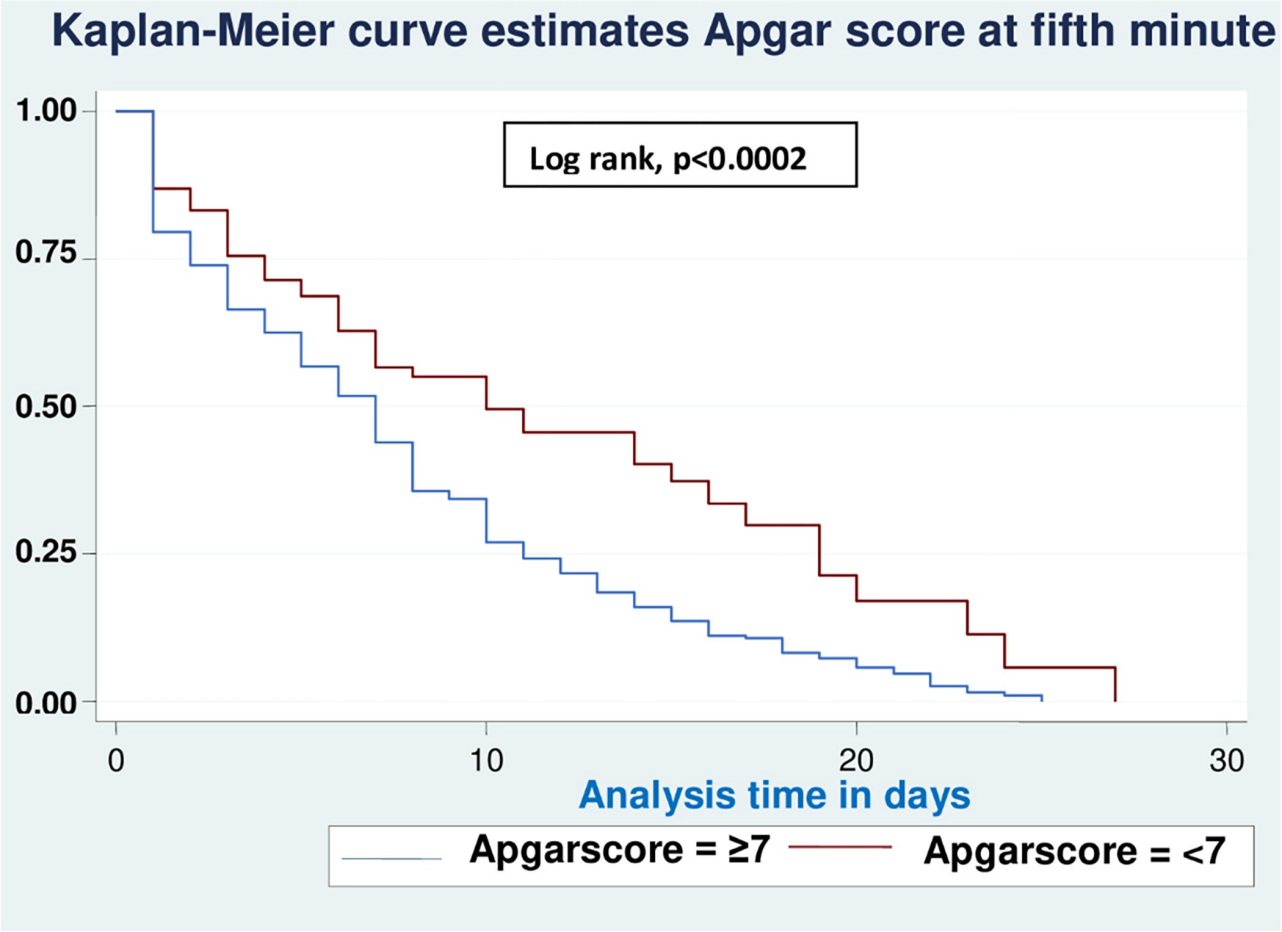
Comparison of Kaplan-Meier survival curves for Apgar score (5min) with the log rank test of neonates with RD admitted to the NICU in UOGCSH, 2021.

Based on various sets of predictors, the Kaplan-Meier survival curve was utilized to determine the overall survival status of neonates with RD. Similar to this, the study found roughly parallel graphs for the length of follow-up when plotting Kaplan Meier survival using gestational age (A), birth weight (B), sepsis (C), hypothermia (D), and Apgar scores for the first (E) and fifth minutes (F), indicating that the proportional hazard assumption was met (**Figs 2–7**).

### Predictors of time to recovery respiratory distress

Following the testing of each variable in turn, the bivariable analyses revealed that residency, neonate age at admission in days, birth weight in grams, gestational age in weeks, Sepsis, meconium aspiration syndrome, jaundice, hypoglycemia, hypothermia, APGAR score at first minute, APGAR score at fifth minute, parity, ANC follow up four times, pregnancy related problems, place of delivery, and multiple pregnancy were all significantly associated with time to recovery of RD.

After entering all of the aforementioned variables into the multi-variable Cox regression mode, very low birth weight (AHR = 0.17, 95% CI: 0.08–0.41), low birth weight (AHR = 0.50, 95% CI: 0.31–0.81), very preterm (AHR = 0.42, 95% CI: 0.20–0.89), sepsis (AHR = 0.50, 95% CI: 0.38–0.65), hypothermia (AHR = 0.61, 95% CI: 0.39–0.81), and Apgar scores less than seven at first (AHR = 0.35, 95% CI: 0.15–0.79) and fifth minute (AHR = 0.45, 95% CI: 0.20–0.97)were significantly associated with the time to recovery of RD (**Table 3**).

**Table 3:** Bivariable and multivariable Cox proportional regression analysis for predictors of time to recovery of respiratory distress in UOGCSH, 2021.

Variables	Recovery from RD		CHR (95% CI)	AHR (95% CI)
	Censored (%)	Event (%)		
Residency				
Urban	68(15%)	183(40.5%)	1	1
Rural	73(16%)	129(28.5%)	1.23(0.98–1.55)	0.93(0.73–1.18)
Neonate age at admission in days				
0–6	132(29.2%)	283(26.6%)	1	1
7–28	9(2%)	28(6.2%)	0.76(0.51–1.11)	0.55(0.37–0.83)
Birth weight in grams				
Normal birth weight	49(10.8%)	180(39.8%)	1	1
Very low birth weight	35(7.7%)	15(3.3%)	0.09(0.05–0.16)**	0.17(0.08–0.41) **
Low birth weight	53(11.7%)	102(22.6%)	0.33(0.26–0.43)**	0.50(0.31–0.81) *
Over birth weight	4(0.9%)	14(3.1%)	0.78(0.45–1.35)	1.01(0.57–1.77)
Gestational age in weeks				
Term	53(11.7%)	176(38.9%)	1	1
very preterm	42(9.3%)	17(3.8%)	0.12(0.07–0.21) **	0.42(0.20–0.89) *
preterm	45(10%)	93(20.6%)	0.36(0.27–0.47) **	0.84(0.51–1.37)
Post term	1(0.2%)	25(5.5%)	1.20(0.79–1.83)	1.32(0.85–2.06)
Sepsis				
No	39(8.6%)	114(25.2%)	1	1
Yes	102(22.6%)	197(43.6%)	0.39(0.31–0.50) **	0.50(0.38–0.65) **
Meconium aspiration syndrome				
No	101(22.3%)	242(53.5%)	1	1
Yes	40(8.8%)	69(15.3%)	1.70(1.29–2.25) **	1.09(0.81–1.46)
Jaundice				
No	125(27.7%)	187(41.4%)	1	1
Yes	16(3.5%)	24(5.3%)	0.77(0.51–1.17)	0.91(0.59–1.41)
Hypoglycemia				
No	92(20.4%)	222(49.1%)	1	1
Yes	49(10.4%)	89(19.7%)	0.60(0.47–0.78) **	0.84(0.64–1.11)
Hypothermia				
No	26(5.8%)	38(8.4%)	1	1
Yes	115(25.4%)	273(60.4%)	0.75(0.53–0.97) **	0.61(0.40–0.81) *
APGAR score at first minute				
≥7	84(18.6%)	264(58.4%)	1	1
<7	60(13.3%)	54(11.9%)	0.53(0.39–0.72) **	0.35 (0.15–0.79) *
APGAR score at fifth minute				
≥7	81(17.9%)	257(56.9%)	1	1
<7	57(12.6%)	47(10.4%)	1.69(1.26–2.27) *	0.45(0.20–0.97) *
Parity				
<5	123(27.2%)	280(61.9%)	1	1
≥5	18(4%)	31(6.9%)	0.75(0.52–1.09)	0.93(0.36–2.36)
Number of ANC follow up				
Less than four times	66(14.6%)	94(20.8%)	1	1
Four and above	75(16.6%)	217(48%)	1.28(1.01–1.63)	1.19(0.91–1.54)
Pregnancy related problems				
No	109(24.1%)	260(57.5%)	1	1
Yes	32(7.1%)	51(11.3%)	0.75(0.55–1.01)	0.79(0.57–1.10)
Place of delivery				
Hospitals	85(18.8%)	250(55.3%)	1	1
Transfer	56(12.4%)	61(13.5%)	1.29(0.97–1.71)	1.21(0.90–1.63)
Multiple pregnancy				
No	117(25.9%)	292(64.6%)	1	1
Yes	24(5.3%)	19(4.2%)	0.58(0.36–0.92) *	0.82(0.51–1.33)

(*) = p-value <0.05, and (**) = p-value ≤0.0001

When compared to normal birth weight, neonates admitted with very low birth weight had83% delay in time to recovery of RD. Likewise, the time to recovery of RD among neonates who had been low birth weight was delayed by 50% as compared to their counterparts. The hazard of prolonged time to recovery from RD was 58% higher in Very preterm neonates than in their counterparts. Neonates with sepsis were delayed by 50% in time to recovery of RD as compared to neonates without sepsis. Similarly, the time to recovery of RD among neonates admitted with hypothermia was prolonged by 39% as compared to its counterparts.

**Fig 8:**
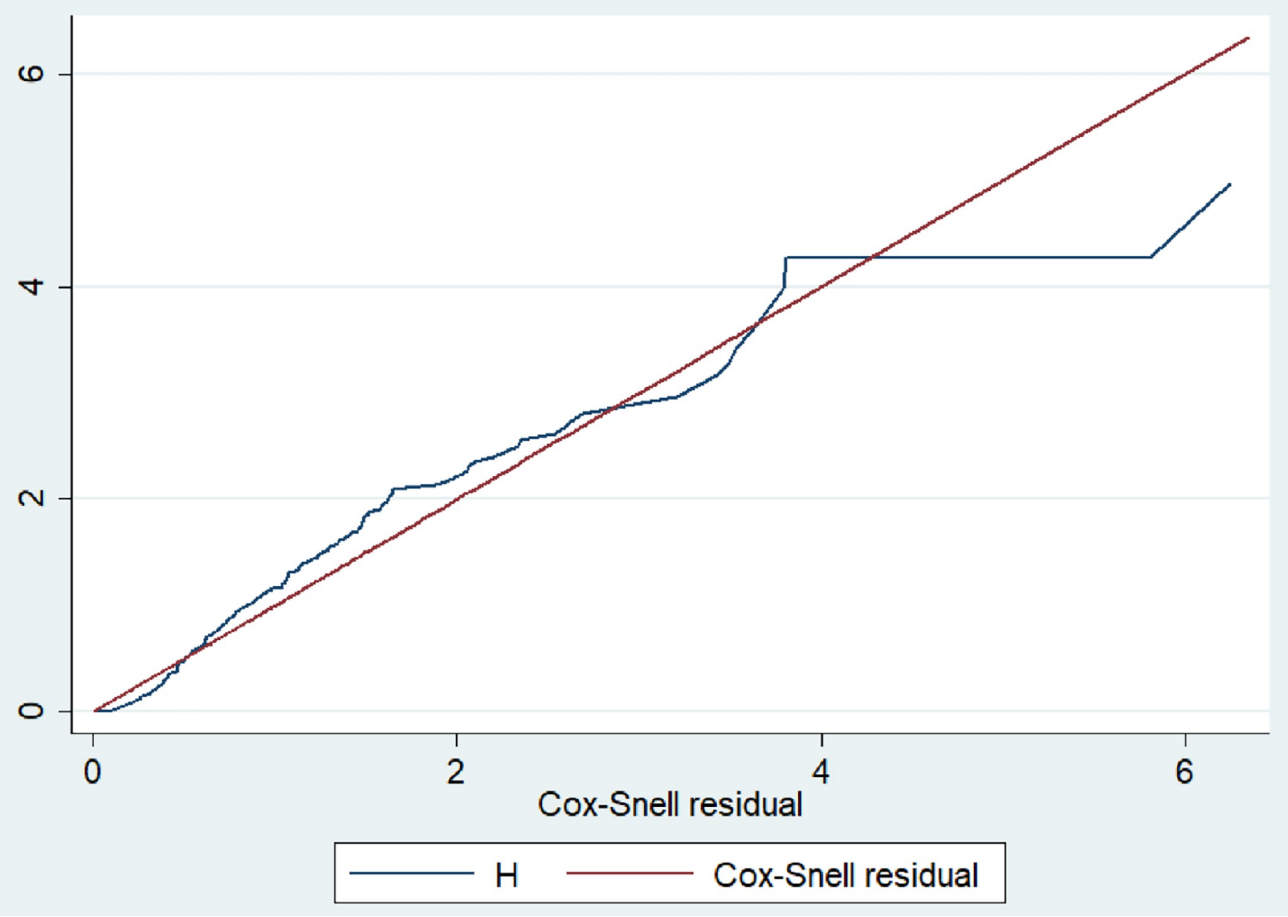
Cox-Snell Residual Graph for the goodness of model fitness that shows the hazard function follows the 45° closed to the baseline, in UOGCSH, 2021.

The recovery time was delayed by 65%thoseneonates who had an Apgar score of less than seven at the first minute compared with neonates having an Apgar score greater than or equal to seven and 55% delayed recovery time at a fifth minute Apgar score of less than seven compared with neonates having an Apgar score greater than or equal to seven. The proportional hazard assumption was checked in the full model using the Schoenfeld residual global test, and it was found to be met (χ2 = 28.42, P-value = 0.0998). Furthermore, the Cox Snell residual test was used to determine the goodness of fit for the fitted model, which revealed that the model was adequate because the Cox-Snell Residual Graph for the goodness of model fitness indicated that the hazard function follows the 45 closed to the baseline **(Fig 8).**

## Discussion

This study evaluated incidence, time to recovery and predictors among neonates admitted with respiratory distress in the University of Gondar comprehensive specialized hospital of neonatal intensive care unit.

In this study, the incidence rate of RD was 11.5%. Studies done in india(2.83%) [8] nepal(4.6%), in saud arbia (1.64%) [22], in sudan(4.83%) [5] weremuch lower lower than our findings.The differences in the study settings, which may offer more sophisticated maternal newborn care services in some locations than in others, may have contributed to the variation seen in these studies. The variations seen between studies may also be caused by sample size, study design, and population socio-demographic characteristics.However, the study participants were term neonates with respiratory distress, whereas our findings were term neonates plus preterms, suggesting that the incidence rate of prematurity has increased.

In this study,the median recovery time from RD was 7 days (IQR = 3–13). This study is in line with the overall median survival recovery time of all neonates of admission in neonatal intensive care units of Dire Dawa l Hospital, which is 7 days.This study shares similarities with it in that it was conducted among neonates admitted to hospitals, the age range,and the sample size was nearly the same, and both confirmed and clinically diagnosed cases were taken into account [23].

Several factors, including very low birth weight, low birth weight, very preterm birth, sepsis, hypothermia, and an Apgar score of less than seven at the first and fifth minutes, were found to be significant predictors of the length of time it took neonates to recover from RD after being admitted to the hospital.

The current study showed that neonates who had very low birth weight had 83% delayed to recovery from RD and that of low birth weight had 50% risk of delayed compared to normal birth weights. These findings are supported by a study performed in South Africa [24] and Uganda [25].The proportion surviving increases as birth weight increases, which means that neonates with low birth weights stayed in hospitals for a longer period of time than those with normal birth weights.

In this study, very preterm neonates admitted with RD showed 58% delayed recovery compared with term neonates. This was supported by the clinical evidence thatas gestational age increases, fetal lung maturity and the production of surfactants will be advanced, and the risk of developing different comorbidities associated with prematurity may increase, which leads to lengthen the admission and elongated the recovery time.

In this study, neonates with sepsis at admission were delayed by 45% to recover from respiratory distress compared to those who had no sepsis. This study is comparable with a study performed in Ethiopia [21]. This is because comorbidity worsens the severity of a disease, which might require long-term admission and prolonged recovery time [26].

This result showed that hypothermic neonates admitted with RD had 39% delayed recovery compared with those not hypothermic with RD. Studies in China [27] and Iran [28] supported this evidence that hypothermia with RD leads to increased oxygen consumption, which leads to hypoxemia, which in turn leads to pulmonary vasoconstriction, the reduced release of pulmonary surfactant and decreased work by respiratory muscles, increasing respiratory distress that delayed recovery. Respiratory distressed neonates with hypothermia, on the other hand, had a lower average gestational age and birth weight, as well as a higher likelihood of having a low Apgar score and an early-onset infection, resulting in a longer recovery time [29].

On the other hand, respiratory distress in neonates is most often associated with hypothermia, especially in very preterm infants, and very low birth weights cause morbidity and prolonged hospitalization because of the double burden [30].

This study reveals that the survival time of neonates was increased for a neonate who had an Apgar score of less than seven at the first and fifth minute compared with those having an Apgar score greater than or equal to seven. The findings are supported by studies performed in the USA [31] and China [32]. The Apgar score, on the other hand, is frequently criticized because it does not accurately identify or predict subsequent acute respiratory disorders and neurodevelopment outcomes in newborns, and many people regard it as out of date. However, until a simpler and more useful scoring system for assessing neonates is developed, the Apgar score remains a valid and rapid index for assessing cardiorespiratory adaptation at birth and the effectiveness of resuscitation, as well as assessing the concurrent health problems of the neonates immediately at birth, which leads to a long recovery time [12].

As an implication, there is a scarcity of data on RD incidence, time to recovery, and predictors. As a result, the discovery could be used to forecast the length of recovery time in neonates with RD. It could also be used to predict the severity of problems in RD identified with predictors of time to recovery and aid in clinical decision making at hospitals. Furthermore, it is prognostic information for clinicians caring for neonates and their families that RD with the identified features may have a longer recovery time as these have economic and social implications on the family, especially in resource-constrained areas.

### Limitations of the study

Since the data were collected from secondary sources, some important predictors, such as socioeconomic factors such as nutritional status of mother, educational level, and birth interval, might be missed, which may have a significant effect on recovery time from RD. The study area covers only UOGCSH; its generalizability to all hospitals of the region and Ethiopia may not be possible, and this might also decrease our precision.

## Conclusion

The incidence and time to recovery in this study were discreetly acceptable as compared to previous study. The predictors that were independently associated with incidence and time to recovery of respiratory distress were very low birth weight, low birth weight, very preterm, sepsis, hypothermia, and Apgar scores less than seven at first and fifth minute. The aforementioned predictors could be used to identify neonates with respiratory distress who are at risk of developing a long-term illness and guide prompt referral to hospitals. This will also provide clinicians with prognostic information, as longer recovery times have economic and social implications in resource limited countries like Ethiopia.

## Supporting information

S1 File This is a check list and questionnaire (English version). (DOCX)

S2 File This is STATA data. (DTA)
