# Supplementary material for: Incidence, time to recovery and predictors among neonates admitted with respiratory distress to the neonatal intensive care unit at the University of Gondar Comprehensive Specialized Hospital, Northwest Ethiopia, 2021
Source: PLoS One. 2022 Dec 15;17(12):e0278887. doi: 10.1371/journal.pone.0278887 (PMC9754192; doi:10.1371/journal.pone.0278887)
Supplement: S1 File — (DOCX) [file pone.0278887.s001.docx]

**Supportive information**

**Table 4: An abstract checklist to determine incidence, time to recovery and predictors of respiratory distress among neonates admitted in neonatal intensive care unit in UOGCSH, Ethiopia, 2021.**

| **Part I: Socio-Demographic Characteristics of both the neonate and the mother** | | | | | | | | |
| --- | --- | --- | --- | --- | --- | --- | --- | --- |
| Serial No | | | Questions | | | Possible answers | |  |
| **Question for the mother** | | | | | |  | |  |
| 101 | | Age | | | |  | |  |
| 102 | | Place of residency | | | | 1. Rural 2. Urban | |  |
| **Question for the neonate** | | | | | |  | |  |
| 103 | | Sex | | | | 1. Male 2. Female | |  |
| 104 | | Age in day | | | |  | |  |
| 105 | | Birth weight in gram | | | |  | |  |
| 106 | | Gestational age at birth in a week | | | |  | |  |
| 107 | | Date of admission | | | |  | |  |
| 108 | | Date of discharge | | | |  | |  |
| **Part 2: A maternal medical condition** | | | | | |  | |  |
| 201 | | Has she has been diagnosed with any medical problem? | | | | 1. Yes 2. No | | If ‘no’ skip Q# 202 |
| 202 | | If the answer yes for question # 201, what was the diagnosis problem | | | | 1. DM 2. HTN 3. HIV/AIDS 4. Other (specify)…. | |  |
| **Part 3: The neonatal medical condition** | | | | | | | | |
| 301 | | Had the neonate been diagnosed with any medical disorders? | | 1. Yes 2. No | | | If ‘no’ skip to Q# 308 | |
| 302 | | Does the neonate have Asphyxia | | 1. Yes 2. No | | |  | |
| 303 | | Does the neonate have Sepsis? | | 1. Yes 2. No | | |  | |
| 304 | | Does the neonate have Hyaline membrane disease? | | 1. Yes 2. No | | |  | |
| 305 | | Does the neonate have RD | | 1. Yes 2. No | | |  | |
| 306 | | Does the neonate have jaundice | | 1. Yes 2. No | | |  | |
| 307 | | Does the neonate have hypoglycemia | | 1. Yes 2. No | | |  | |
| 308 | | Newborns temperature within 1hr of admission | |  | | |  | |
| 309 | | APGAR score | | 1. 1^st^ minute_______ 2. 5^th^ minute______ | | |  | |
| 310 | | Length of hospital stay in a day | |  | | |  | |
| 311 | | Patient status | | 1. Death 2. Alive 3. Referred 4. Other…. | | |  | |
| 312 | | Time of neonatal death/Discharge | |  | | |  | |
| **Part 4: Obstetric and Gynecological Predictors** | | | | | | | | |
| 401 | Number of gravidities | | | |  | |  | |
| 402 | Number of parity | | | |  | |  | |
| 403 | Does the mother had ANC follow up | | | | 1. Yes 2. No | |  | |
| 404 | Which among the following do you have diagnosed? | | | | 1. PROM 2. Preeclampsia 3. Other…... | |  | |
| 405 | What was her Current mode of delivery? | | | | 1. SVD 2. CS 3. Instrumental | |  | |
| 406 | Place of delivery | | | | 1. Home 2. Health center 3. Hospitals | |  | |
| 407 | Was the current pregnancy multiple | | | | 1. Yes 2. No | |  | |
